# Reducing ED Visits: Optimizing an Interdisciplinary Telemedicine Program in Singapore Nursing Homes

**DOI:** 10.1093/geroni/igaf122.1255

**Published:** 2025-12-31

**Authors:** Jiameng Yuan, Gayathri Devi Nadarajan, Marcus Ong, Jean Mui Hua Lee, Hanzhang Xu

**Affiliations:** Duke University, Durham, North Carolina, United States; Singapore General Hospital, Singapore, Singapore; Duke-NUS Medical School, Singapore, Singapore; Sengkang General Hospital, Singapore, Singapore; Duke University, Durham, North Carolina, United States

## Abstract

Emergency department (ED) utilization among nursing home (NH) residents is reportedly high, with one study showing 42.4% of visits leading to hospital admission. This could be particularly pronounced in rapidly aging societies like Singapore. Prior research has suggested that many ED visits from NHs residents were are often preventable. Our prior work has developed and pilot tested a telemedicine program (EAGLEcareACT [Enhancing Advance Care Planning, Geriatrics, and End-of-Life Care Acute Care Team]) that connected ED physicians with NH staff to triage patients from NHs and address care needs within NHs. Preliminary evaluation has suggested EAGLEcareACT reduces avoidable ED visits among older adults from NHs. To broadly implement EAGLEcareACT through a large-scale, pragmatic trial, we sort to conduct a qualitative pre-implementation study, guided by the Consolidated Framework for Implementation Research (CFIR). We plan to conduct semi-structured qualitative interviews with 2 NH managers, 4 NH staff, 2 ED physicians, and 4 family members of NH residents. We will comprehensively assess the current practice at each targeted NH and the complexity of care needs for NHs residents. We will explore perceived barriers and facilitators of this interdisciplinary telemedicine program and seek input on person-centered outcomes that should be measured in this pragmatic trial. All interviews will be recorded and transcribed verbatim, with fieldnotes supplementing the interview transcription. We will analyze data in Nvivo 14 using both deductive/inductive coding based on CFIR domains. This information would optimize the implementation and expansion of EAGLEcareACT as well as enhance its sustainability and effectiveness in NH settings.

